# Syncope and Collapse Are Associated with an Increased Risk of Cardiovascular Disease and Mortality in Patients Undergoing Dialysis

**DOI:** 10.3390/ijerph15102082

**Published:** 2018-09-21

**Authors:** Shih-Ting Huang, Tung-Min Yu, Tai-Yuan Ke, Ming-Ju Wu, Ya-Wen Chuang, Chi-Yuan Li, Chih-Wei Chiu, Cheng-Li Lin, Wen-Miin Liang, Tzu-Chieh Chou, Chia-Hung Kao

**Affiliations:** 1 Division of Nephrology, Taichung Veterans General Hospital, Taichung 407, Taiwan; kitheroborn@hotmail.com (S.-T.H.); yu5523@gmail.com (T.-M.Y.); wmj530@gmail.com (M.-J.W.); colaladr@yahoo.com.tw (Y.-W.C.); 2 Graduate Institute of Public Health, China Medical University, Taichung 404, Taiwan; 3 Graduate Institute of Biomedical Sciences and School of Medicine, College of Medicine, China Medical University, Taichung 404, Taiwan; 4 Division of Nephrology, Ministry of Health and Welfare Chiayi Hospital, Chiayi 600, Taiwan; nightrider731@msn.com; 5 Graduate Institute of Clinical Medical Science, China Medical University, Taichung 404, Taiwan; D17550@mail.cmuh.org.tw (C.-Y.L.); givychiu@gmail.com (C.-W.C.); 6 Department of Anesthesiology, China Medical University Hospital, Taichung 404, Taiwan; 7 Management Office for Health Data, China Medical University Hospital, Taichung 404, Taiwan; orangechengli@gmail.com; 8 College of Medicine, China Medical University, Taichung 404, Taiwan; 9 Graduate Institute of Biostatistics, China Medical University, Taichung 404, Taiwan; wmliang@mail.cmu.edu.tw; 10 Department of Public Health, China Medical University, Taichung 404, Taiwan; 11 Department of Health Risk Management, College of Public Health, China Medical University, Taichung 404, Taiwan; 12 Department of Nuclear Medicine and PET Center, China Medical University, Taichung 404, Taiwan; 13 Department of Bioinformatics and Medical Engineering, Asia University, Taichung 404, Taiwan

**Keywords:** syncope and collapse, acute coronary syndrome, dialysis

## Abstract

*Objective*: This study explored the impact of syncope and collapse (SC) on cardiovascular events and mortality in patients undergoing dialysis. *Methods*: Patients undergoing dialysis with SC (*n* = 3876) were selected as the study cohort and those without SC who were propensity score-matched at a 1:1 ratio were included as controls. Major adverse cardiovascular events (MACEs), including acute coronary syndrome (ACS), arrhythmia or cardiac arrest, stroke, and overall mortality, were evaluated and compared in both cohorts. *Results*: The mean follow-up periods until the occurrence of ACS, arrhythmia or cardiac arrest, stroke, and overall mortality in the SC cohort were 3.51 ± 2.90, 3.43 ± 2.93, 3.74 ± 2.97, and 3.76 ± 2.98 years, respectively. Compared with the patients without SC, those with SC had higher incidence rates of ACS (30.1 vs. 24.7 events/1000 people/year), arrhythmia or cardiac arrest (6.75 vs. 3.51 events/1000 people/year), and stroke (51.6 vs. 35.7 events/1000 people/year), with higher overall mortality (127.7 vs. 77.9 deaths/1000 people/year). The SC cohort also had higher risks for ACS, arrhythmia or cardiac arrest, stroke, and overall mortality (adjusted hazard ratios: 1.28 (95% confidence interval (CI) = 1.11–1.46), 2.05 (95% CI = 1.50–2.82), 1.48 (95% CI = 1.33–1.66), and 1.79 (95% CI = 1.67–1.92), respectively) than did the non-SC cohort. *Conclusion*: SC was significantly associated with cardiovascular events and overall mortality in the patients on dialysis. SC may serve as a prodrome for cardiovascular comorbidities, thereby assisting clinicians in identifying high-risk patients.

## 1. Introduction

Patients undergoing dialysis have an extremely high prevalence of death (198 deaths/1000 patients/year) [1]. Among such patients, cardiovascular disease (CVD) is the major cause of death, accounting for approximately 40% of all-cause mortality [2]. In the United States Renal Data System database, the leading specific cause of cardiac death is arrhythmia or sudden cardiac arrest (SCA), accounting for the deaths of approximately 60% of patients, followed by coronary heart disease (CHD), accounting for the deaths of approximately 20% of patients in the dialysis population [3]. Observational studies have shown a graded association between a decreased glomerular filtration rate and the risks of death and major adverse cardiovascular events (MACEs) in community-based populations [4,5]. Thus, morbidity and mortality due to MACEs can occur from the early stages of chronic kidney disease, even before the development of end-stage renal disease (ESRD) [5]. Although the current trend of declining mortality rates among patients with ESRD [6] can be explained by the secondary prevention of CVD [7], little evidence of benefits generated by risk factor modification has been found among a dialysis population. Lipid modification with statin treatment has no benefits in terms of CVD mortality, and antiplatelet therapy reduces the risk of myocardial infarction (MI) but not CVD mortality in the dialysis population [8,9]. The most likely explanation for ineffective treatment is that most cardiac deaths are attributed to SCA or arrhythmias, and the responsible mechanisms are still unclear. For the treatment and prevention of SCA in patients undergoing dialysis, the use of beta blockers is recommended in patients with heart failure due to systolic dysfunction [10], and angiotensin II receptor blockers may provide protection against CVD mortality [11]. Although these evidence-based therapies may partially explain the slow, steady decline in the overall cardiac mortality and SCA rate [2], the dismal outcomes and limited treatment of CHD and SCA in patients undergoing dialysis remain a challenge for nephrologists.

Syncope, defined as the transient loss of consciousness with rapid and spontaneous recovery, has been associated with acute and chronic renal failure [12,13]. The clinical scenario of patients undergoing dialysis experiencing syncope and collapse (SC) during or after dialysis is not uncommon. In such patients, syncope is a presenting symptom of an underlying cardiac disease (aortic stenosis and coronary artery disease (CAD)), dysrhythmia (electrolyte disturbance and QT changes), pulmonary hypertension, and intradialytic or postural hypotension [14,15,16,17,18]. However, whether clinical correlations between such medical conditions and SC can translate into CVD risk in a dialysis population remains unclear. Despite the advanced knowledge of evaluation and management of syncope in a general population [12], no large-scale study has examined whether syncope is associated with the clinical outcomes of CVD in a dialysis population [19]. This study investigated the characteristics and prognosis of SC in a specialized population.

## 2. Materials and Methods

### 2.1. Data Sources

This retrospective cohort study used the Registry for Catastrophic Illness Patient Database (RCIPD), a subset of the National Health Insurance Research Database, which is under the National Health Insurance (NHI) program. Taiwan launched the single-payer compulsory NHI program in 1995, and ever since, the program has covered over 99% of the population of Taiwan (23.74 million individuals in 2015) [20]. In the NHI program, an insurant with a major disease such as ESRD can apply to the Registry for Catastrophic Illness Patients, which exempts a patient from copayments for his or her medical services. The details of the RCIPD have been described in previous studies [21,22]. Disease definitions in the RCIPD are based on the International Classification of Diseases, Ninth Revision, Clinical Modification (ICD-9-CM) diagnostic codes. The Ethics Review Board of China Medical University Hospital in Taiwan approved this study (CMUH104-REC2-115-CR2).

### 2.2. Study Design and Participants

We identified patients with incident ESRD (ICD-9-CM code 585) between 1 January 1998 and 31 December 2011, and divided them into two cohorts: an SC (ICD-9-CM code 780.2) cohort and a non-SC comparison cohort. The index date was defined as the date of SC diagnosis and the corresponding non-SC comparison patients were followed from a randomly assigned date in the same year. Patients who were younger than 18 years, had undergone renal transplantation (ICD-9-CM codes V42.0 and 996.81), or had developed acute coronary syndrome (ACS; ICD-9-CM codes 410, 411.1, and 411.8), arrhythmia or cardiac arrest (ICD-9-CM codes 427.1, 427.4, 427.5, 798.1 and 798.2), or stroke (ICD-9-CM codes 430–438) before the index date were excluded from both cohorts. We used a 1:1 propensity score (PS) matching to balance baseline characteristics between the two cohorts. Baseline characteristics including age, dialysis modality, sex, and comorbidities (namely deep vein thrombosis, pulmonary embolism, valvular heart disease, atrial fibrillation, diabetes mellitus (DM), hypertension, and hyperlipidemia), were used to determine predicted disease probability obtained through logistic regression. We further classified the patients on dialysis into hemodialysis (HD) and peritoneal dialysis (PD) groups based on the dialysis modality at day 90 after the first dialysis session.

### 2.3. Outcomes and Relevant Variables

ACS (ICD-9-CM codes 410, 411.1 and 411.8), arrhythmia or cardiac arrest (ICD-9-CM codes 427.1, 427.4, 427.5, 798.1, and 798.2), stroke (ICD-9-CM codes 430–438), and overall mortality events were defined as the primary endpoint. All patients were followed from the index date to the date when the endpoint occurred or until the end of 2011. Pre-existing comorbidities that could have affected the primary outcome, namely deep vein thrombosis (ICD-9-CM code 453.8), pulmonary embolism (ICD-9-CM code 415.1), valvular heart disease (ICD-9-CM codes 394–397, 398.9 and 424.0–424.3), atrial fibrillation (ICD-9-CM code 427.31), DM (ICD-9-CM code 250), hypertension (ICD-9-CM codes 401–405), and hyperlipidemia (ICD-9-CM code 272), were investigated. These comorbidities had been diagnosed in previous inpatient claims records or in at least three successive outpatient claim records before the index date. We included SC-related medical visits as defined by outpatient visits or hospital admissions. For each patient in the SC cohort, annual medical visits were calculated based on the number of SC-related medical visits during the entire follow-up period divided by the number of follow-up person-years.

### 2.4. Statistical Analysis

The Chi-squared test was used to examine the difference between the distributions of demographic status and comorbidities in categorical variables between the SC and non-SC cohorts. The Student’s *t* test was used to examine the continuous variables. The cumulative incidence of endpoints (including ACS, arrhythmia or cardiac arrest, stroke, and overall mortality) was calculated using the Kaplan–Meier method, and the log-rank test was used to examine differences between the two cohorts. Univariable and multivariable Cox proportional hazard regression models were used to estimate hazard ratios (HRs) and 95% confidence intervals (CIs) to evaluate the risks of endpoints. Estimations were performed as crude and adjusted for possible confounders. Considering death as a competing risk factor for other CVD outcomes, we performed a competing risk survival analysis by using the subdistribution hazard function. We further assessed the effects of the dose response of SC-related medical visits on the risks of endpoints based on the number of outpatient visits and hospitalizations for SC. A *p*-value of less than 0.05 was considered significant for all tests. All statistical analyses was performed using SAS, version 9.4 (SAS Institute, Inc., Cary, NC, USA).

## 3. Results

### 3.1. Incidence and Prevalence of Syncope in the Dialysis Population

A total of 132,404 patients with incident dialysis were identified between 1998 and 2011 from the Registry for Catastrophic Illness Patients. The incidence and prevalence rates of SC among the patients undergoing dialysis increased during the follow-up period (Supplemental Figure S1). In 2011, the incidence and prevalence rates of syncope were 7.05% and 10.8%, respectively. The incidence and prevalence rates of SC were higher among patients with underlying hypertension and DM than in those without.

### 3.2. Patient Characteristics

The SC cohort contained 3876 patients. The patients in the non-SC cohort were selected as controls after PS matching at a 1:1 ratio (Table 1). The two cohorts were similar in terms of demographic characteristics and comorbidities. In both cohorts, approximately 51% of the patients were elderly (≥65 years) and approximately 48% were men. In the SC cohort, the proportion of patients with ESRD who received HD (90.1%) was higher than that who received PD. The major comorbid disease in both cohorts was hypertension (88.2% vs. 88.5%), followed by hyperlipidemia (40.1% vs. 40.2%), and DM (39.9% vs. 40.8%).

### 3.3. Risk Factors for Primary Outcomes

Table 2 lists the predictors of ACS, arrhythmia or cardiac arrest, stroke, and overall mortality in the PS-matched cohorts. Among these comorbidities and demographic conditions, the presence of SC and DM were universal risk factors for ACS, arrhythmia or cardiac arrest, stroke, and overall mortality. Men had higher risks of ACS and stroke, and had higher overall mortality than did women, and the risks of ACS, arrhythmia or cardiac arrest, stroke, and overall mortality increased with age. Valvular heart disease was associated with the risk of ACS and higher overall mortality. Atrial fibrillation was associated with the risk of arrhythmia or cardiac arrest, stroke, and higher overall mortality. Hypertension was associated with the risk of ACS and stroke. The patients with hyperlipidemia had a higher risk of ACS.

### 3.4. Primary Outcomes and Risk Stratification

**Figure 1:**
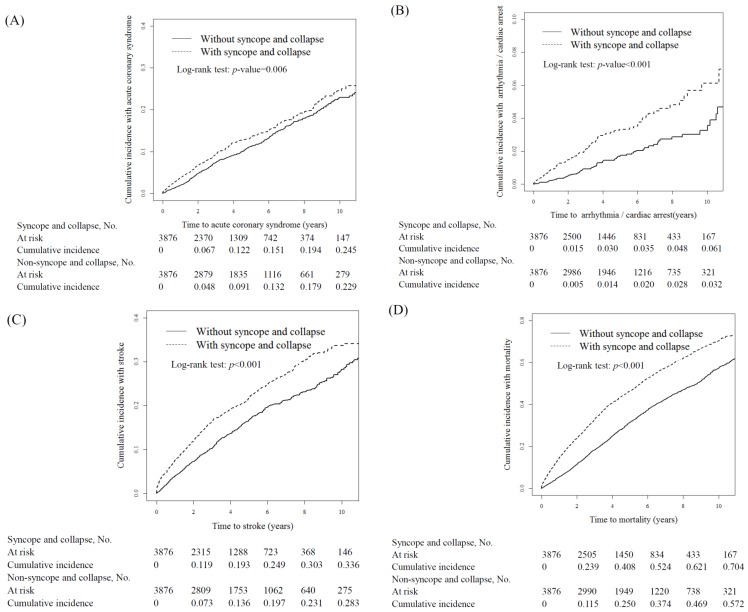
Cumulative incidence between patients undergoing dialysis with and without SC for the events of (**A**) ACS; (**B**) arrhythmia or cardiac arrest; (**C**) stroke; and (**D**) mortality.

In the SC cohort, the mean follow-up periods until the occurrence of the primary endpoints, namely ACS, arrhythmia or cardiac arrest, stroke, and overall mortality, were 3.51 ± 2.90, 3.43 ± 2.93, 3.74 ± 2.97, and 3.76 ± 2.98 years, respectively. Figure 1 shows that the cumulative incidence of ACS, arrhythmia or cardiac arrest, stroke, and overall mortality was significantly higher in the SC cohort than in the non-SC cohort (log-rank test, *p* < 0.001). In both cohorts, the incidence rate of outcome events was highest for overall mortality, followed by stroke, ACS, and arrhythmia or cardiac arrest (Table 3). Overall, the incidence rates of ACS (30.1 vs. 24.7 events/1000 people/year), arrhythmia or cardiac arrest (6.75 vs. 3.51 events/1000 people/year), stroke (51.6 vs. 35.7 events/1000 people/year), and overall mortality (127.7 vs. 77.9 deaths/1000 people/year) were higher in the SC cohort than in the non-SC cohort, with adjusted HRs (aHRs) of 1.28 (95% CI = 1.11–1.46), 2.05 (95% CI = 1.50–2.82), 1.48 (95% CI = 1.33–1.66), and 1.79 (95% CI = 1.67–1.92), respectively. The sex-specific SC cohort-to-non-SC cohort relative risks of ACS, arrhythmia or cardiac arrest, stroke, and overall mortality were considerably higher for women and men. The incidence density rates of ACS, arrhythmia or cardiac arrest, stroke, and overall mortality increased with age in both cohorts. The age-specific SC cohort-to-non-SC cohort relative risk of ACS was higher in the age group of ≤49 years. The age-specific SC cohort-to-non-SC cohort relative risk of arrhythmia or cardiac arrest was higher in the age groups of 50–64 and ≥65 years. The age-specific SC cohort-to-non-SC cohort relative risks of stroke and overall mortality were higher in all age groups. The incidence density rates of ACS, arrhythmia or cardiac arrest, stroke, and overall mortality were higher among the patients who received HD than among those who received PD in both cohorts. The dialysis modality-specific SC cohort-to-non-SC cohort relative risks of ACS, arrhythmia or cardiac arrest, stroke, and overall mortality were significantly higher among the patients who received HD than among those who received PD. The patients with comorbidities in the SC cohort had considerably higher risks of ACS, arrhythmia or cardiac arrest, stroke, and overall mortality than did those with comorbidities in the non-SC cohort. The risks of ACS, arrhythmia or cardiac arrest, and stroke did not significantly differ between the patients without comorbidities in the two cohorts. However, the SC cohort-to-non-SC cohort relative risk of mortality was higher among the patients without comorbidities in the SC cohort. The SC cohort-to-non-SC cohort relative risk of stroke was significant in all stratified patients except those without comorbidities. The risk of overall mortality in all stratifications remained higher in the SC cohort than in the non-SC cohort. Based on the competing risk analysis results, the patients with SC still had higher risks of arrhythmia or cardiac arrest and stroke, with adjusted sub-hazard ratios of 1.72 (95% CI = 1.26–2.35) and 1.29 (95% CI = 1.16–1.44), than did the patients without SC (Supplemental Table S1).

Graded based on the frequency of medical visits, the effects of SC severity on the outcome risk were shown in Table 4. The patients with SC who had more than three SC-related medical visits per year exhibited a 4.57-fold increased risk of ACS (95% CI = 3.74–5.60) compared with those without SC. Similar results were observed for arrhythmia or cardiac arrest and stroke. The risk of overall mortality increased up to 4.86 (95% CI = 4.40–5.38) among those with SC who had three or more medical visits compared with those without SC (trend test, *p* < 0.001).

## 4. Discussion

This is the first retrospective cohort study to report a significant association between SC and MACEs or mortality. Among the patients with SC, the mean follow-up period until the occurrence of MACEs or mortality was approximately 3.5 years, indicating an ominous disease course. The presence of SC is independently associated with an increased risk of MACEs or mortality, regardless of age, sex, dialysis modality, or comorbidities. Increased frequencies of medical visits for SC episodes multiplied the risks of CVD and mortality. Given the extremely high mortality events and prevalent underlying comorbid conditions of patients undergoing dialysis, the importance of the relationship between SC and MACEs or mortality cannot be overlooked.

Despite high mortality among the dialysis population, the clinical epidemiology of CVD (incidence and prevalence) remains unreported. Patients undergoing dialysis are characterized by a high prevalence of occult and/or silent myocardial ischemia [23,24], which has been associated with increased risks of MI, arrhythmia, and sudden cardiac death (SCD) [25]. The atypical symptoms of CVD may delay its diagnosis and adversely affect the outcomes [26]. Current guidelines recommend assessing for CVD in all patients at the initiation of dialysis regardless of symptoms [27]. However, despite this, specific guidelines for the evaluation of CAD [28], arrhythmia, and SCA in patients undergoing dialysis remain limited because of the complex pathophysiology of these conditions. 

Patients undergoing dialysis have a higher risk of stroke than does the general population [29]. Older age, male sex, diabetes, and hypertension were independent risk factors for ischemic and hemorrhagic strokes in patients undergoing dialysis [29]. Until now, the preventive effect of anticoagulants on the risk of stroke was not evident, and the prerequisite issue of stroke risk assessment from atrial fibrillation in patients undergoing dialysis has rarely been discussed [30,31]. In our study, a high incidence of stroke was noted in the SC cohort, and SC was associated with an increased risk of stroke regardless of sex, age, dialysis modality, or comorbidities (Table 3). We identified SC as an independent predictor for incident stroke, and this provided us with helpful insights into risk assessment among the dialysis population.

Most patients with SCA have no premonitory symptoms, and if such symptoms are present, they are usually nonspecific, rendering SCA the most fatal form of CVD among patients undergoing dialysis. Interacting risk factors such as comorbid coronary artery disease, myocardial abnormalities, electrolyte abnormalities, and heart failure are complex and underlie the increased incidence of SCA among the dialysis population [32]. Although we could identify patients with a high risk of SCA, the optimal approach for preventing SCA in patients undergoing dialysis is unclear and varies with risk stratification. Implantable cardioverter defibrillator (ICD) placement was associated with a significant reduction in the risk of death in patients undergoing dialysis who had survived ventricular fibrillation or cardiac arrest and is recommended as a secondary method of preventing SCA in such patients [33]. However, the estimated one-year mortality after ICD implantation remained high (approximately 40%) [2], and ICD therapy is apparently underutilized in patients undergoing dialysis [33]. These facts illustrate the predicaments faced by the medical field related to the prevention and management of CVD among the dialysis population. Identifying early symptoms to predict high-risk patients seems the most practical method of managing CVD.

In the general population, cardiac syncope may be related to CVD morbidity and mortality [13,34]. Most associated deaths are related to concomitant underlying comorbidities rather than syncope [35]. In our study, the patients with comorbid conditions outnumbered those without comorbidities in the SC cohort. This indicated that comorbidities are prevalent in patients with SC undergoing dialysis, and the increased CVD and mortality risks in the SC cohort may be closely related to underlying comorbidities. By contrast, reflex and orthostatic syncope accounts for most syncopal episodes (30.6%) and represents a benign disease course in the general population [34]. In our study, the presence of SC did not increase the risks of ACS, arrhythmia or cardiac arrest, and stroke in patients undergoing dialysis who had no underlying comorbidities (Table 3). This suggests that SC in such patients could be largely related to non-cardiac syncope. However, in such patients, the presence of SC increased the risk of overall mortality (Table 3). This might be explained based on consequences of syncope; for example, falling and accidents among patients undergoing dialysis [36,37]. Nevertheless, further research is required to verify this causality. 

Regarding dialysis modality and patient outcomes, PD may provide relative short-term survival benefits but comparable or reduced survival after the first few years compared with HD [38,39]. Our study results showed that PD exhibited relative benefits in terms of MACEs and survival compared with HD during the follow-up period (Table 3). In the PD and HD patients, the presence of SC considerably increased the risk of mortality, with aHRs of 2.17 and 1.66 (95% CI = 1.65–2.86 and 1.55–1.78), respectively. In the non-SC cohort, the incident rate of mortality was considerably lower among the patients who underwent PD than among those who underwent HD (54.2 vs. 80.0/1000 people/year). However, in the SC cohort, the survival rate among patients undergoing PD approached that among those undergoing HD (111.9 vs. 129.4/1000 people/year). This result indicated that the presence of SC is an independent predictor of mortality, regardless of dialysis modality. Although selection bias and differences in terms of comorbid diseases may have occurred among patients undergoing different dialysis modalities, this did not affect our result regarding the effect of SC on survival.

Our study is the first nationwide study on syncope in patients undergoing dialysis and included a large cohort of patients with a long follow-up duration. The results validated the clinical impact of SC on adverse CVD outcomes and mortality in a dialysis population. Evaluating the patients who presented with SC enabled the early detection and management of underlying diseases. Secondary prevention of MACEs might improve outcomes. Despite their strengths, the results of this study must be viewed with consideration of the following limitations. First, the patients were retrospectively identified based on diagnostic codes, which may have led to the underestimation of the true incidence in both cohorts. Second, unpredictable confounding factors may influence the outcomes of an observational study. To overcome this limitation, we used PS matching to balance the confounding factors among the cohorts, thereby mitigating the inherent difference at baseline. However, the retrospective design established the association but not the causality between exposure and outcomes. Third, the patients with SC might have not received an immediate presumptive diagnosis in their initial medical visits. In a previous study, only approximately 50% of SC cases received a definite diagnosis [12] among the general population. Evaluating the causes of SC among the patients undergoing dialysis in this study was more difficult, and the causes may be multifactorial, including orthostatic change, hypovolemia, cardiac or pulmonary diseases, and antihypertensive medication [12,19,40]. Because of these inherent limitations, additional studies are warranted to examine the interaction between the cause of syncope and underlying diseases in patients undergoing dialysis.

## 5. Conclusions

This study demonstrated that as a prodrome, SC can be an effective predictor of incident MACEs and mortality, both of which have practical implications in the timely evaluation of patients undergoing dialysis.

## Figures and Tables

**Table 1:** Comparison of demographics and comorbidities between dialysis patients with and without syncope and collapse (SC).

Syncope and Collapse			
Characteristics	No (*n* = 3876)	Yes (*n* = 3876)	*p*-Value
	*n* (%)	*n* (%)	
Age, years			0.89
≤49	585 (15.1)	599 (15.5)	
50–64	1300 (33.5)	1301 (33.6)	
≥65	1991 (51.4)	1976 (51.0)	
Mean (SD) ^#^	63.6 (13.2)	63.7 (13.3)	0.91
Dialysis modality			0.003
Peritoneal dialysis	310 (8.00)	384 (9.91)	
Hemodialysis	3566 (92.0)	3492 (90.1)	
Gender			0.86
Female	2039 (52.6)	2031 (52.4)	
Male	1837 (47.4)	1845 (47.6)	
Comorbidity			
Deep vein thrombosis	228 (5.88)	228 (5.88)	0.99
Pulmonary embolism	5 (0.13)	5 (0.13)	0.99
Valvular heart disease	534 (13.8)	542 (14.0)	0.79
Atrial fibrillation	260 (6.71)	260 (6.71)	0.99
Diabetes	1581 (40.8)	1547 (39.9)	0.43
Hypertension	3432 (88.5)	3417 (88.2)	0.60
Hyperlipidemia	1559 (40.2)	1553 (40.1)	0.89

Chi-squared test; ^#^: Student’s *t* test.

**Table 2:** Hazard ratios (HRs) of outcomes in association with sex, age, and comorbidities in univariate and multivariate Cox regression models.

Variable	ACS		Arrhythmia/Cardiac Arrest		Stroke		Mortality	
	Crude HR (95% CI)	Adjusted HR ^†^ (95% CI)	Crude HR (95% CI)	Adjusted HR ^†^ (95% CI)	Crude HR (95% CI)	Adjusted HR ^†^ (95% CI)	Crude HR (95% CI)	Adjusted HR ^†^ (95% CI)
Syncope and collapse	1.21 (1.06, 1.39) **	1.28 (1.11, 1.46) ***	1.93 (1.41, 2.65) ***	2.05 (1.50, 2.82) ***	1.40 (1.26, 1.56) ***	1.48 (1.33, 1.66) ***	1.65 (1.54, 1.77) ***	1.79 (1.67, 1.92) ***
Gender (Men vs. women)	1.21 (1.05, 1.38) **	1.24 (1.08, 1.42) **	1.24 (0.91, 1.68)	-	1.23 (1.11, 1.38) ***	1.20 (1.08, 1.34) ***	1.18 (1.11, 1.27) ***	1.19 (1.11, 1.27) ***
Age, years	1.03 (1.02, 1.03) ***	1.02 (1.02, 1.03) ***	1.03 (1.01, 1.04) ***	1.03 (1.01, 1.04) ***	1.03 (1.03, 1.04) ***	1.03 (1.03, 1.04) ***	1.05 (1.04, 1.05) ***	1.05 (1.04, 1.05) ***
Dialysis modality								
Peritoneal dialysis	1 (Reference)	1 (Reference)	1 (Reference)	1 (Reference)	1 (Reference)	1 (Reference)	1 (Reference)	1 (Reference)
Hemodialysis	1.32 (1.01–1.73) *	1.09 (0.83, 1.44)	1.43 (0.75, 2.71)	-	1.46 (1.16, 1.83) **	1.14 (0.91, 1.44)	1.23 (1.08, 1.41) **	0.86 (0.75, 0.98) *
Baseline comorbidities (yes vs. no)								
Deep vein thrombosis	1.25 (0.96, 1.63)	-	0.97 (0.49, 1.89)	-	0.78 (0.60, 1.01)	-	1.10 (0.95, 1.26)	-
Pulmonary embolism	2.46 (0.61, 9.86)	-	-	-	1.43 (0.36, 5.71)	-	1.02 (0.38, 2.71)	-
Valvular heart disease	1.39 (1.16, 1.68) ***	1.30 (1.08, 1.58) **	1.37 (0.90, 2.09)	-	1.17 (1.00, 1.37) *	-	1.33 (1.21, 1.46) ***	1.15 (1.05, 1.27) **
Atrial fibrillation	1.54 (1.18, 2.01) **	1.25 (0.95, 1.64)	2.59 (1.58, 4.24) ***	2.23 (1.35, 3.67) **	1.51 (1.23, 1.87) ***	1.26 (1.02, 1.56) *	2.13 (1.89, 2.40) ***	1.65 (1.46, 1.86) ***
Diabetes	1.88 (1.64, 2.16) ***	1.62 (1.40, 1.87) ***	1.84 (1.35, 2.51) ***	1.76 (1.28, 2.41) ***	1.90 (1.71, 2.13) ***	1.77 (1.58, 1.99) ***	1.52 (1.42, 1.63) ***	1.51 (1.41, 1.62) ***
Hypertension	1.99 (1.55, 2.57) ***	1.44 (1.11, 1.87) **	2.06 (1.14, 3.71) *	1.57 (0.86, 2.87)	1.98 (1.61, 2.44) ***	1.55 (1.25, 1.91) ***	1.28 (1.15, 1.43) ***	0.98 (0.88, 1.09)
Hyperlipidemia	1.62 (1.42, 1.86) ***	1.38 (1.19, 1.59) ***	1.29 (0.95, 1.77)		1.18 (1.06, 1.32) **	0.97 (0.86, 1.09)	1.00 (0.93, 1.07)	-

Crude HR: relative hazard ratio; ACS: acute coronary syndrome; 95% CI: 95% confidence interval; ^†^ Only confounding variables that were found to be significant in the univariable model were further analyzed; * *p* < 0.05, ** *p* < 0.01, *** *p* < 0.001.

**Table 3:** Incidence and adjusted HRs of ACS, arrhythmia or cardiac arrest, stroke, and mortality based on sex, age, and comorbidities between patients undergoing dialysis with and without SC.

Variables	Syncope and Collapse						Compared to Control	
	No			Yes				
	Events *n*	PY	Rate ^#^	Events *n*	PY	Rate ^#^	Crude HR (95% CI)	Adjusted HR ^†^ (95% CI)
ACS								
All	435	17,613	24.7	409	13,611	30.1	1.21 (1.06, 1.39) **	1.28 (1.11, 1.46) ***
Gender								
Female	217	9601	22.6	204	7450	27.4	1.20 (0.99, 1.46)	1.26 (1.04, 1.53) *
Male	218	8011	27.2	205	6161	33.3	1.22 (1.01, 1.48) *	1.29 (1.06, 1.56) *
*p*-value for interaction								0.97
Age, years								
≤49	32	3421	9.36	56	2930	19.1	2.16 (1.39, 3.36) ***	2.30 (1.48, 3.57) ***
50–64	155	6205	25.0	146	5030	29.0	1.16 (0.92, 1.45)	1.23 (0.98, 1.54)
≥65	248	7987	31.1	207	5650	36.6	1.19 (0.99, 1.43)	1.17 (0.97, 1.41)
*p*-value for interaction								0.16
Dialysis modality								
Peritoneal dialysis	26	1413	18.4	31	1319	23.5	1.28 (0.76, 2.16)	1.30 (0.76, 2.21)
Hemodialysis	409	16,200	25.3	378	12,292	30.8	1.21 (1.05, 1.39) **	1.24 (1.08, 1.43) **
*p*-value for interaction								0.86
Comorbidity ^§^								
No	21	1746	12.0	14	1282	10.9	0.91 (0.46, 1.79)	1.02 (0.51, 2.01)
Yes	414	15,867	26.1	395	12,329	32.0	1.23 (1.07, 1.41) **	1.27 (1.10, 1.46) ***
*p*-value for interaction								0.41
Arrhythmia/cardiac arrest								
All	65	18,542	3.51	98	14,527	6.75	1.93 (1.41, 2.65) ***	2.05 (1.50, 2.82) ***
Gender								
Female	31	10,067	3.08	49	7900	6.20	2.01 (1.28, 3.16) **	2.08 (1.32, 3.27) **
Male	34	8475	4.01	49	6627	7.39	1.85 (1.19, 2.87) **	2.03 (1.30, 3.16) **
*p*-value for interaction								0.76
Age, years								
≤49	5	3526	1.42	5	3089	1.62	1.16 (0.33, 4.02)	1.13 (0.32, 3.92)
50–64	25	6561	3.81	38	5375	7.07	1.87 (1.13, 3.11) *	1.99 (1.20, 3.31) **
≥65	35	8456	4.14	25	6063	9.07	2.23 (1.45, 3.42) ***	2.24 (1.46, 3.43) ***
*p*-value for interaction								0.49
Dialysis modality								
Peritoneal dialysis	4	1457	2.74	6	1381	4.35	1.71 (0.48, 6.14)	1.73 (0.48, 6.29)
Hemodialysis	61	17,085	3.57	92	13,146	7.00	1.96 (1.42, 2.71) ***	2.01 (1.45, 2.78) ***
*p*-value for interaction								0.75
Comorbidity ^§^								
No	5	1783	2.80	4	1322	3.03	1.07 (0.29, 3.99)	1.16 (0.31, 4.38)
Yes	60	16,760	3.58	94	13,205	7.12	2.00 (1.45, 2.77) ***	2.08 (1.50, 2.88) ***
*p*-value for interaction								0.37
Stroke								
All	611	17,114	35.7	686	13,304	51.6	1.40 (1.26, 1.56) ***	1.48 (1.33, 1.66) ***
Gender								
Female	298	9341	31.9	338	7294	46.3	1.42 (1.21, 1.66) ***	1.49 (1.28, 1.75) ***
Male	313	7773	40.3	348	6010	57.9	1.39 (1.19, 1.62) ***	1.46 (1.25, 1.71) ***
*p*-value for interaction								0.92
Age, years								
≤49	55	3384	16.3	71	2935	24.2	1.50 (1.05, 2.13) *	1.56 (1.10, 2.22) *
50–64	176	6104	28.8	226	4871	46.4	1.59 (1.30, 1.93) ***	1.70 (1.40, 2.08) ***
≥65	380	7627	49.8	389	5498	70.8	1.36 (1.18, 1.56) ***	1.37 (1.19, 1.58) ***
*p*-value for interaction								0.52
Dialysis modality								
Peritoneal dialysis	23	1376	16.7	57	1279	44.6	2.44 (1.50, 3.96) ***	2.46 (1.51, 4.00) ***
Hemodialysis	588	15,738	37.4	629	12,025	52.3	1.36 (1.22, 1.53) ***	1.40 (1.25, 1.57) ***
*p*-value for interaction								0.01
Comorbidity ^§^								
No	36	1686	21.4	25	1265	19.8	0.89 (0.54, 1.49)	0.98 (0.58, 1.64)
Yes	575	15,428	37.3	661	12,039	54.9	1.43 (1.28, 1.60) ***	1.48 (1.32, 1.66) ***
*p*-value for interaction								0.09
Mortality								
All	1447	18,569	77.9	1859	14,555	127.7	1.65 (1.54, 1.77) ***	1.79 (1.67, 1.92) ***
Gender								
Female	715	10,078	71.0	942	7910	119.1	1.70 (1.54, 1.87) ***	1.82 (1.65, 2.01) ***
Male	732	8491	86.2	917	6645	138.0	1.60 (1.46, 1.77) ***	1.78 (1.61, 1.96) ***
*p*-value for interaction								0.45
Age, years								
≤49	99	3527	28.1	160	3093	51.7	1.86 (1.45, 2.40) ***	1.93 (1.50, 2.48) ***
50–64	367	6572	55.8	543	5390	100.7	1.82 (1.60, 2.08) ***	1.90 (1.66, 2.17) ***
≥65	981	8470	115.8	1156	6072	190.4	1.70 (1.56, 1.85) ***	1.71 (1.57, 1.86) ***
*p*-value for interaction								0.19
Dialysis modality								
Peritoneal dialysis	79	1459	54.2	155	1385	111.9	2.02 (1.54, 2.65) ***	2.17 (1.65, 2.86) ***
Hemodialysis	1368	17,110	80.0	1704	13,170	129.4	1.63 (1.52, 1.75) ***	1.66 (1.55, 1.78) ***
*p*-value for interaction								0.09
Comorbidity ^§^								
No	101	1783	56.7	134	1321	101.4	1.75 (1.35, 2.26) ***	2.07 (1.59, 2.69) ***
Yes	1346	16,786	80.2	1725	13,233	130.4	1.64 (1.53, 1.76) ***	1.75 (1.63, 1.88) ***
*p*-value for interaction								0.48

PY: person/year; Rate ^#^: incidence rate per 1000 people/year; Crude HR: relative hazard ratio; ^†^ Only confounding variables that were found to be significant in the multivariable model were further analyzed. ^§^: comorbidity; patients with any one of the following: diabetes, hypertension, hyperlipidemia, deep vein thrombosis, pulmonary embolism, and valvular heart disease were classified into the comorbidity group. * *p* < 0.05, ** *p* < 0.01, *** *p* < 0.001.

**Table 4:** Risks of ACS, arrhythmia or cardiac arrest, stroke, and mortality among patients with SC based on frequency of medical visits for SC in Cox proportional hazard regression.

Frequency for Medical Visit, Per Year	Event	PY	Rate ^#^	Crude HR (95% CI)	Adjusted HR ^†^ (95% CI)
ACS					
Without syncope and collapse	435	17,613	24.7	1.00	1.00
With syncope and collapse					
0–1	218	11,461	19.0	0.78 (0.66, 0.91) **	0.83 (0.71, 0.98) *
1–2	61	1049	58.2	2.41 (1.84, 3.17) ***	2.26 (1.72, 2.97) ***
≥3	130	1101	118.0	4.85 (3.97, 5.93) ***	4.57 (3.74, 5.60) ***
*p*-value for trend					<0.001
Arrhythmia/cardiac arrest					
Without syncope and collapse	65	18,542	3.51	1.00	1.00
With syncope and collapse					
0–1	51	11,926	4.28	1.23 (1.86, 1.78)	1.35 (0.93, 1.95)
1–2	16	1191	13.4	4.03 (2.32, 7.02) ***	3.83 (2.19, 6.67) ***
≥3	31	1409	22.0	6.55 (4.23, 10.1) ***	6.08 (3.92, 9.42) ***
*p*-value for trend					
Stroke					
Without syncope and collapse	611	17,114	35.7	1.00	1.00
With syncope and collapse					
0–1	332	11,379	29.2	0.82 (0.71, 0.93) **	0.88 (0.77, 1.01)
1–2	112	977	114.6	2.99 (2.43, 3.66) ***	2.82 (2.30, 3.47) ***
≥3	242	948	255.3	6.47 (5.55, 7.54) ***	6.26 (5.36, 7.30) ***
*p*-value for trend					<0.001
Mortality					
Without syncope and collapse	1447	18,569	77.9	1.00	1.00
With syncope and collapse					
0–1	1000	11,944	83.7	1.09 (1.00, 1.18) *	1.22 (1.13, 1.33) ***
1–2	307	1193	257.4	3.48 (3.07, 3.94) ***	3.24 (2.86, 3.67) ***
≥3	552	1418	389.3	5.22 (4.72, 5.76) ***	4.86 (4.40, 5.38) ***
*p*-value for trend					<0.001

PY: person/year; Rate ^#^: incidence rate per 1000 people/year; Crude HR: relative hazard ratio; ^†^ Only confounding variables that were found to be significant in the multivariable model were further analyzed; * *p* < 0.05, ** *p* < 0.01, *** *p* < 0.001.

## Supplementary Materials

The following are available online at http://www.mdpi.com/1660-4601/15/10/2082/s1: Table S1: Subhazard ratios (SHRs) of ACS, arrhythmia or cardiac arrest, and stroke among patients undergoing dialysis with and without SC estimated using the competing-risk regression models, Figure S1: Trends in syncope incidence and prevalence rate per 1000/people/year based on the underlying comorbidities of DM and hypertension (HTN) in the Taiwanese dialysis population, 1998–2011.

## Abbreviations

SC: syncope and collapse; MACEs: major adverse cardiovascular events; ACS: acute coronary syndrome; CVD: cardiovascular disease; ESRD: end-stage renal disease; CHD: coronary heart disease; MI: myocardial infarction; HR: hazard ratio; CI: confidence interval; ICD-9-CM: International Classification of Diseases, Ninth Revision, Clinical Modification; NHI: National Health Insurance; LHID: Longitudinal Health Insurance Database.
