# Supplementary material for: Syncope and Collapse Are Associated with an Increased Risk of Cardiovascular Disease and Mortality in Patients Undergoing Dialysis
Source: Int J Environ Res Public Health. 2018 Sep 21;15(10):2082. doi: 10.3390/ijerph15102082 (PMC6210976; doi:10.3390/ijerph15102082)
Supplement: Supplementary file 1 [file ijerph-15-02082-s001.pdf]

Syncope and Collapse are Associated with an Increased Risk of Cardiovascular Disease and Mortality in Patients Undergoing Dialysis

Shih-Ting Huang, Tung-Min Yu, Tai-Yuan Ke, Ming-Ju Wu, Ya-Wen Chuang, Chi-Yuan Li, Chih-Wei Chiu, Cheng-Li Lin, Wen-Miin Liang, Tzu-Chieh Chou and Chia-Hung Ka

Table S1. Subhazard ratios (SHRs) of ACS, arrhythmia or cardiac arrest, and stroke among patients undergoing dialysis with and without SC estimated using the competing-risk regression models.

Outcome event	Syncope and collapse	
	No	Yes
ACS		
Crude SHR (95% CI)	1(Reference)	1.06(0.93, 1.21)
Adjusted SHR† (95% CI)	1(Reference)	1.07(0.94, 1.23)
Arrhythmia / cardiac arrest		
Crude SHR (95% CI)	1(Reference)	1.69(1.24, 2.31) ***
Adjusted SHR† (95% CI)	1(Reference)	1.72(1.26, 2.35) ***
Stroke		
Crude SHR (95% CI)	1(Reference)	1.27(1.14, 1.41) ***
Adjusted SHR† (95% CI)	1(Reference)	1.29(1.16, 1.44) ***

SHR: relative subhazard ratio; † Only confounding variables that were found to be significant in the multivariable model were further analyzed; * $p < 0.05$, ** $p < 0.01$, *** $p < 0.001$.

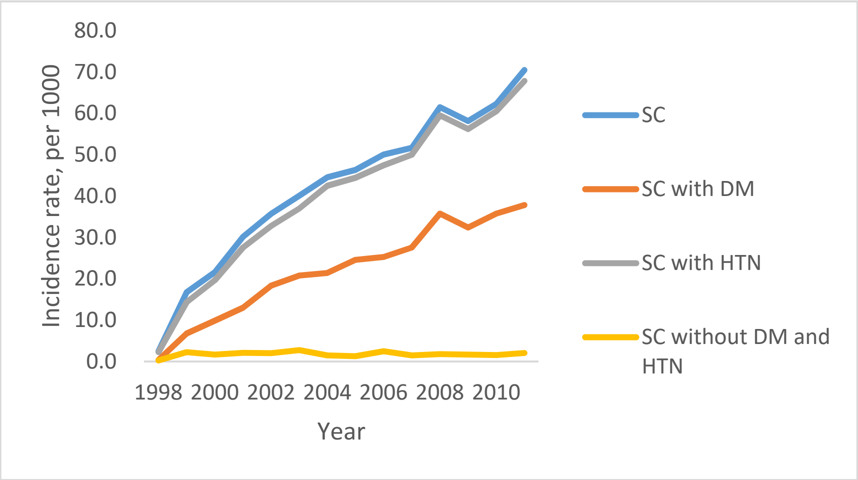

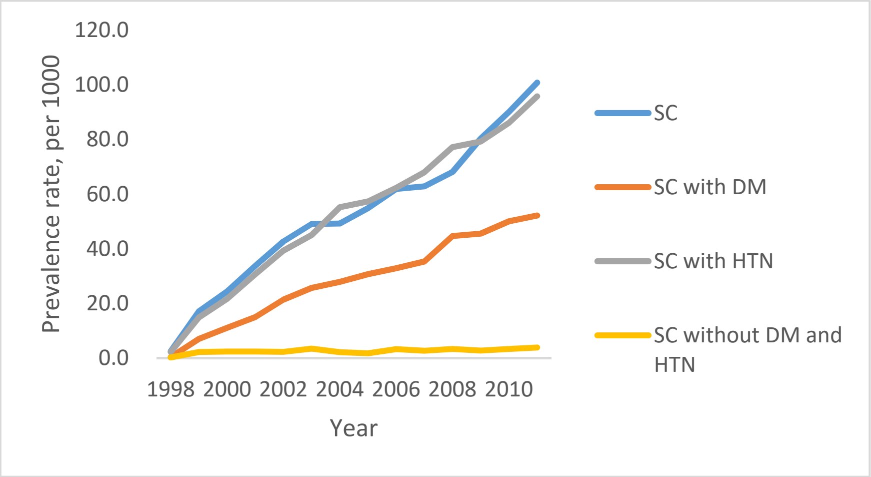

Figure S1. Trends in syncope incidence and prevalence rate per 1000/people/year based on the underlying comorbidities of DM and hypertension (HTN) in the Taiwanese dialysis population, 1998–2011.
